# Target-specific modulation of cortical narrowband gamma by basal ganglia nuclei: a case report

**DOI:** 10.3389/fnhum.2026.1851996

**Published:** 2026-08-05

**Authors:** Mallory L. S. Eisel, Jackson N. Cagle, Kara A. Johnson, Filipe Sarmento, Hikaru Kamo, Doris D. Wang, Joshua K. Wong, Philip A. Starr, Coralie de Hemptinne

**Affiliations:** 1 Department of Neurology, University of Florida, Gainesville, FL, United States; 2 College of Medicine, Florida State University, Tallahassee, FL, United States; 3 Norman Fixel Institute for Neurological Diseases, University of Florida, Gainesville, FL, United States; 4 Department of Neurology, Juntendo University School of Medicine, Tokyo, Japan; 5 Department of Neurological Surgery, Weill Institute for Neurosciences, University of California, San Francisco, San Francisco, CA, United States

**Keywords:** case report, Parkinson’s disease (PD), deep brain stimulation (DBS), electrocorticography (ECOG), norrow band gamma (NBG), globus pallidus internus and extrenus (GPi-e), subthalamic nucleus (STN)

## Abstract

**Introduction:**

Narrow-band gamma (NBG) is an oscillatory activity in the gamma band (60–90 Hz) that has been observed in the subthalamic nucleus (STN), the motor cortex, and, more recently, the globus pallidus internus (GPi) of Parkinson’s disease (PD) patients, often in the on-medication state. Therapeutic DBS has been shown to modulate NBG, often entraining cortical and subcortical NBG to half the stimulation frequency, often referred to as finely tuned gamma (FTG). However, the differential effect of STN, GPi, and GPe DBS on cortical NBG has never been explored in the same patient.

**Method:**

We report a case of a 54-year-old female patient with early-onset PD harboring *PRKN* variants, who underwent GPi-DBS implantation to supplement the effect of a prior STN lead. During surgery, cortical potentials were recorded via a temporary electrocorticography (ECoG) strip placed over the sensorimotor cortex while sequentially stimulating the STN, GPi, and GPe using the same DBS parameters (bipolar mode, 3 V, 180 Hz, 60 μs). Given the severity of symptoms, the participant took medication before surgery. Therefore, all recordings were done in the on-medication state in the presence of leg dyskinesia.

**Result:**

At baseline, the primary motor cortex (M1) exhibited prominent NBG activity at 68 Hz. Both STN and GPi DBS induced similar effects, leading to NBG suppression. These neurophysiological changes were accompanied by modest clinical improvements in rigidity and dystonia. In contrast, GPe DBS did not suppress NBG and was not associated with any clinical benefit.

**Discussion:**

Our results show that therapeutic and nontherapeutic basal ganglia stimulation differentially modulated M1 NBG, supporting the potential utility of this activity as a biomarker for therapeutic stimulation. The absence of entrainment during STN and GPi stimulation might be due to high frequency (180 Hz) exceeding the range of cortical synchronization and FTG emergence.

## Introduction

Parkinson’s disease (PD) is the fastest-growing neurological disorder worldwide (Dorsey et al., 2018), characterized by bradykinesia, rigidity, or rest tremor (Postuma et al., 2015). Deep brain stimulation (DBS) targeting the subthalamic nucleus (STN) or globus pallidus internus (GPi) is an established treatment for PD, providing sustained motor improvement and reducing motor fluctuation (Ramirez-Zamora and Ostrem, 2018). Recording local field potentials (LFP) from the DBS electrode or electrocorticography (ECoG) from electrodes placed over the cortex has provided a better understanding of the pathophysiology underlying PD (Crowell et al., 2012). Growing evidence suggests that abnormal neural synchronization correlates with PD motor symptoms. The off-medication hypokinetic state is characterized by excessive basal ganglia beta band activity (13–30 Hz) (Brown and Williams, 2005) that correlates with the severity of bradykinesia and rigidity (Kuhn et al., 2006), while narrow band gamma (NBG, 60–90 Hz) is found in in the STN (Brown et al., 2001), the primary motor cortex (M1) (Swann et al., 2016), and the GPi (Kamo et al., 2026b) of PD patients, most often in the ‘on’ medication state, though it has been observed off medication when DBS was applied (Wiest et al., 2021; Kamo et al., 2026a). Stimulation has been shown to modulate NBG, shifting it from its native frequency and often entraining it to half the stimulation frequency, referred to as finely tuned gamma (FTG) (Swann et al., 2016; Oehrn et al., 2024). Interestingly, this entrainment depends on stimulation parameters, and higher frequencies, such as 180 Hz, do not always produce reliable locking. These oscillations have emerged as a potential biomarker of the therapeutic ‘on’ state (Swann et al., 2016; Kamo et al., 2026b; Swann et al., 2018; Wiest et al., 2022) and for physiology-guided DBS (Gilron et al., 2021). Although basal ganglia DBS is known to alter M1 activity, the distinct effects of STN, GPi, and GPe stimulation on cortical activity within the same individual have not yet been investigated.

To investigate the role of basal ganglia stimulation on cortical NBG activity, we report the first-in-human case of sequential STN, GPi, and GPe stimulation with simultaneous cortical field potential recording.

## Case description

### Methods

#### Patient

A 54-year-old female with genetically confirmed Parkinson’s disease (Parkin variant) was enrolled before undergoing left GPi-DBS implantation at the University of California at San Francisco (UCSF). Her symptoms began with dystonia at age 7. She received bilateral STN-DBS (model 3,389, Medtronic, Minneapolis, MN, USA) at age 42 to manage motor fluctuations, followed by a left lead revision at 44 due to lead fracture. Despite initial benefit, her condition progressively worsened, leading to severe rigidity, dystonia, medication-induced dyskinesia, and the inability to walk without assistance when she was referred to UCSF. The persistence of these symptoms prompted the implantation of an additional DBS lead (model 3,387, Medtronic, Minneapolis, MN, USA) in the left GPi at 54 years old. At the time of surgery, the patient’s medication regimen consisted of levodopa/carbidopa (450 mg daily) and trihexyphenidyl (8 mg daily), and the STN-DBS stimulation setting was (R: 9-C+, 3.9 V, 60us, 180 Hz; L: 1-C+, 2.9 V, 60us, 180 Hz). The preoperative Unified Parkinson’s Disease Rating Scale (UPDRS) part III score was 77 in the medication-off/STN-DBS stimulation-off state and 42 in the medication-on/STN-DBS stimulation-on state.

#### Electrode placement and localization

The GPi-DBS lead was implanted based on a method previously described (Starr, 2002). The GPi was identified on a preoperative Magnetic Resonance Imaging (MRI) and verified by microelectrode recordings during the surgery. The lead was placed with 2 contacts within the GPi and 2 contacts in the GPe. In addition, a six-contact ECoG strip (Ad-Tech) was temporally inserted under the dura through the burr hole used for the DBS lead placement and advanced in the direction of the intended target location, the arm area of the primary motor cortex (M1), about 3 cm from the midline and slightly medial to the hand knob, as previously described (Crowell et al., 2012). An intraoperative imaging Computed Tomography (CT) scan was done to confirm the correct placement of the DBS lead and the ECoG strip (Crowell et al., 2012). The CT was then used offline, merged to the preoperative MRI, and nonlinearly registered to overlay segmentations of the STN, GPi, and GPe from the DISTAL atlas (Ewert et al., 2018). The ECoG contacts covering M1 (C4–5) were identified with intraoperative imaging and somatosensory evoked potentials (SSEP) as previously described (Crowell et al., 2012).

#### Cortical recordings

The ECoG potentials were recorded from each contact, referenced to a scalp needle, and sampled at 22 kHz using the Neuro Omega device (Alpha Omega, Nazareth, Israel). Due to symptom severity, the patient continued taking her medications on schedule, leading up to the procedure. Recordings started 3 h after the patient had taken medication, and when experiencing dyskinesia. To study the effect of DBS on M1 activity, cortical field potentials were recorded while the patient was resting with eyes open, while sequentially delivered in a bipolar configuration, from contacts localized in the STN (1–2+, active contact MNI coordinates: 13.5, −14.8, −6.9), GPi (0–1+, active contact MNI coordinates: 23.4, −7.6, −8.8), and GPe (2–3+, active contact MNI coordinates: 23.3, −5.3, −2.3), using identical parameters across all sites (3 V, 180 Hz, 90 μs). For each stimulation condition, ECoG was recorded for at least 30 s before, during, and after DBS. The same stimulation settings were used across all targets to allow direct comparison and were based on STN chronic stimulation parameters. GPe stimulation was used as an internal control for non-therapeutic effects relative to STN and GPi.

#### Signal processing and statistical analysis

Data processing and statistical analyses were performed using custom scripts in MATLAB R2025a (MathWorks, Natick, MA, USA). All cortical recordings were bipolar referenced to the adjacent contact and down-sampled to 1KHz. The 60 Hz powerline noise was removed by performing a third-order Butterworth filter. The spectral power for time-frequency analysis was calculated with the short-time Fourier Transform (STFT) with a 2-s Hamming window (0.5 Hz frequency resolution) and 50% overlap for each time-epoch. The average power spectral density (PSD) for each condition was calculated by taking the average STFT within each condition. In each recording condition, the maximum power within 62-90 Hz was identified, and the corresponding frequency and amplitude were extracted at each STFT period. The drift in NBG frequency was computed by performing thresholding across each condition with an empirical threshold of 10 μV^2^/Hz. A similar analysis was then performed, averaging power between 88 and 92 Hz to determine whether FTG emerged during DBS. Changes in power across conditions were then analyzed using a non-parametric Wilcoxon rank-sum test, with multiple comparisons corrected using the Bonferroni method. All *p*-values reported are after Bonferroni correction. Statistical significance was set at *p* < 0.05. All data are expressed as median (IQR).

#### Participant consent and data availability

Participant provided written consent in agreement with the Declaration of Helsinki and the UCSF institutional review board. Anonymized data will be made available upon request.

## Results

### Localization of DBS leads and ECoG strip

**Figure 1:**
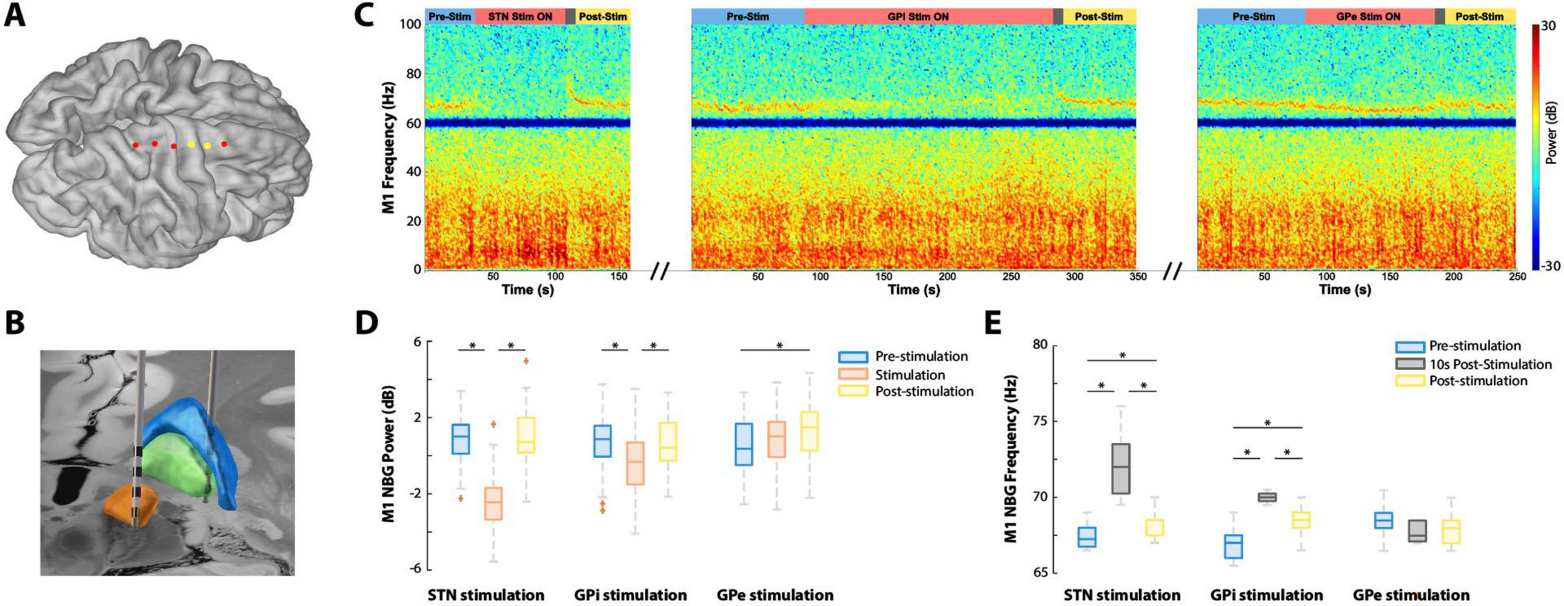
**(A)** ECoG strip placement over the primary motor cortex (M1). Each dot indicates an electrode contact, with contacts 1 and 6 being more posterior and anterior, respectively. Contacts 4 and 5, shown in yellow, were covering the motor cortex. **(B)** DBS leads placement. Localization of the DBS leads and individual contacts (with 0 and 3 being the most ventral and dorsal, respectively) relative to the anatomy of each target structure (orange: STN, green: GPi, blue: GPe). **(C)** Spectrogram over time showing M1 activity before (blue rectangle), during (red rectangle) and after DBS (yellow rectangle). Stimulation was sequentially delieverd from STN (left), GPi (middle) and GPe (right) stimulation using the same stimulation parameters (180 Hz, 3 V, 60 μs). A reduction of NBG was observed during STN and GPi DBS, while GPe stimulation did not modulate NBG power. **(D)** Box plot shows M1 FTG Power (dB) across stimulation status. Blue: before DBS, red: during DBS, yellow: after DBS. Left: STN DBS, middle: GPi DBS, right: GPe DBS. Asterisks denote significance (*p* < 0.01). **(E)** Box plot shows M1 FTG Frequency (Hz) across stimulation status. Blue: before DBS, red: during DBS, yellow: after DBS. Left: STN DBS, middle: GPi DBS, right: GPe DBS. Asterisks denote significance (*p* < 0.01).

Each electrode contact was localized to the corresponding segmented brain structure using the intraoperative CT registered to the preoperative MRI, and to the DISTAL. The contacts 0 and 1 of the GP DBS lead localized in GPi, while contacts 2 and 3 were in the GPe. For the STN lead, contact 1 is localized in the dorsal STN, with contact 2 dorsal to STN. These bipolar contacts were used to deliver stimulation in each structure. Contacts 4 and 5 of the ECoG strip covered the primary motor cortex (Figures 1A,B).

### Effects of STN, GPi, and GPe stimulation on NBG power

To evaluate the effect of basal ganglia DBS on cortical NBG, we computed the PSD from all contact pairs, before, during, and after each stimulation condition, extracted the NBG amplitude, and compared across conditions. The changes in M1 activity induced by stimulation in STN, GPi, and then GPe is shown on the spectrograms over time in Figure 1C. An NBG peak can be observed at ~68 Hz in the DBS off conditions, both before and after delivering DBS (shown in blue and yellow rectangles on Figures 1C–E). Consistent with previous studies, only contact pairs with contacts covering M1 exhibited NBG. Delivering DBS in the STN (1–2+, 180 Hz, 3 V, 60 μs) induced the suppression of NBG activity (Figure 1C red rectangle), which was associated with a slight improvement in rigidity and dystonia based on the patient’s self-report and the brief assessment with passive upper limb movement. The NBG power during stimulation was significantly reduced compared with the non-stimulation periods (both before and after stimulation, Wilcoxon rank-sum test with Bonferroni correction; *p* < 0.001, Figure 1D), whereas no significant difference was found between before and after stimulation (*p* > 1.0). Similarly, GPi stimulation (0–1+, 180 Hz, 3 V, 60 μs) also significantly suppressed NBG activity and was accompanied by mild improvement in rigidity and dystonia (Figures 1C,D; Wilcoxon rank-sum test with Bonferroni correction; p < 0.001). No significant difference was observed when comparing before and after GPi stimulation (Wilcoxon rank-sum test with Bonferroni correction; *p* = 0.58). In contrast, GPe stimulation (2–3+, 180 Hz, 3 V, 60 μs) did not significantly modulate NBG power (Wilcoxon rank-sum test with Bonferroni correction; *p* = 0.64, *p* = 0.09, and *p* > 1.0, respectively, Figures 1C,D). GPe DBS was not associated with symptom improvement. To further examine potential entrainment at half the stimulation frequency, we repeated the same analysis, focusing on 90 Hz (Wilcoxon rank-sum test with Bonferroni correction; all values p > 1.0). No significant changes were observed during DBS, confirming the absence of FTG in this study. Note that leg dyskinesia was present throughout the surgery but was not consistently assessed.

### Temporal analysis of NBG frequency changes

We further investigated the temporal impact of DBS on the frequency of these oscillations by identifying the frequency of the NBG peak using a 1 s moving window. The temporal analysis revealed a stable NBG peak centered at 68 Hz, which quickly disappeared upon STN DBS activation. Interestingly, following cessation of DBS, the NBG reemerged at a higher frequency, ~80 Hz, and gradually returned to its native frequency (68 Hz, Figure 1C). The NBG frequency averaged over the first 10 s after DBS was statistically higher than before and after STN and DBS (grey, Figures 1C,E). A similar trend was observed following GPi stimulation, with suppression of NBG but no entrainment during GPi DBS. Changes induced by GPi DBS were also slightly delayed compared to STN. In contrast, GPe DBS did not suppress NBG (Figure 1E). Thus, 180 Hz stimulation may transiently disrupt M1 NBG and move it away from its excessive pattern, with a gradual return to its intrinsic oscillatory state after stimulation ends.

## Discussion

In this first-in-human study, we investigated the effects of STN, GPi, and GPe DBS on cortical synchronization, focusing on gamma activity in M1 (60–90 Hz). We found that both STN and GPi stimulation similarly modulated NBG, reducing its power during stimulation, although the STN effect was more immediate, possibly through the hyperdirect pathway. This suppression was associated with modest improvements in rigidity and bradykinesia, suggesting a potential link between NBG modulation and therapeutic benefit. In contrast, GPe stimulation did not reduce NBG activity and was not associated with clinical improvement.

Previous studies have shown NBG activity in M1 is commonly present in the on-medication state, and stimulation in the basal ganglia often entrained these oscillations to half the stimulation frequency, referred to as finely tuned gamma (FTG) (Swann et al., 2016; Oehrn et al., 2024; Mathiopoulou et al., 2025; Olaru et al., 2024). In this case report, we observed a stable NBG peak at 68 Hz in the absence of stimulation, consistent with prior findings. However, during DBS, NBG was not entrained but instead reduced. The absence of entrainment during DBS may be due to the stimulation frequency used, 180 Hz, which likely exceeds the physiological range for effective cortical entrainment. The absence of entrainment at 180 Hz is consistent with the Arnold tongue framework, which limits entrainment to a specific frequency range (<180 Hz), and with prior studies demonstrating NBG entrainment using 110–160 Hz (Swann et al., 2016; Oehrn et al., 2024; Mathiopoulou et al., 2025; Sermon et al., 2023). In our study, an entrainment would also require an upward shift toward 90 Hz, thus approximately 22 Hz above the native oscillatory frequency, which may fall outside the entrainment zone. Prior studies have also found that entrainment emerges with specific amplitudes (Oehrn et al., 2024; Mathiopoulou et al., 2025; Sermon et al., 2023). Here, stimulation was delivered at 3 V, which may not have been sufficient to fully entrain the cortical activity.

The modulation of NBG during stimulation differed between basal ganglia targets. We found that therapeutic stimulation of the STN and GPi reduced NBG activity in M1, whereas nontherapeutic stimulation of the GPe did not alter NBG power. This differential effect of GPi versus GPe stimulation is consistent with prior findings in dystonia (Cernera et al., 2025). This contrast suggests that modulation of M1 NBG may be a feature of effective DBS and could serve as a marker of therapeutic engagement. We further found that STN DBS produced a more robust and temporally precise suppression of NBG compared to GPi. This difference may reflect underlying anatomical connectivity, with the STN directly connected to M1 via the hyperdirect pathway, enabling rapid cortical modulation, while the absence of direct projections between GPi and M1 might limit its influence on cortical activity (Nambu et al., 2023).

Chronic adaptive DBS (aDBS) offers a promising therapeutic approach for PD in which stimulation is adjusted in real time based on neuronal signals (Neumann et al., 2019). While beta-band activity has been more explored as the feedback signal, FTG has emerged as a promising biomarker given its predictable entrainment to half the stimulation frequency (Swann et al., 2016; Gilron et al., 2021; Olaru et al., 2024). DBS might induce network changes time-locked to the stimulation pulses, resulting in entrainment at half the stimulation frequency, and potentially strengthening cortico-basal ganglia temporal coordination. Recent studies suggest that FTG-based aDBS may outperform conventional DBS (cDBS), offering greater motor improvement and reduced side effects (Oehrn et al., 2024). Our findings are consistent with prior work showing that therapeutic DBS modulates NBG activity, reinforcing its influence on cortical dynamics. However, we did not observe FTG during stimulation at 180 Hz, likely because this frequency exceeds the physiological range required for entrainment. Our findings highlight the importance of selecting stimulation parameters that align with the intrinsic dynamics of cortical networks when considering FTG as a feedback signal for aDBS.

This study has several limitations. Although FTG has previously been linked to dyskinesia and prokinetic states (Swann et al., 2016; Gilron et al., 2021; Mathiopoulou et al., 2025; Olaru et al., 2024), our study does not allow conclusions about this relationship, given that dyskinesia was not systematically assessed during surgery. Motor symptoms were also evaluated intraoperatively by an unblinded clinician without standardized rating scales. We used the same stimulation settings across all targets to allow direct comparison; these parameters were not optimized for each target and likely differ from the optimal settings, particularly for GP. Although notes from the initial programming indicate a slight improvement in arm relaxation with contact 1 at 2.5 V, there was no formal UPDRS assessment of GPi DBS benefit after DBS implantation, which limits our ability to correlate the intraoperative findings to chronic DBS effects. The ECoG strip was placed over the arm area, whereas targeting the leg motor cortex would likely have better captured the cortical activity related to the observed leg dyskinesia. Additionally, only one stimulation frequency (180 Hz) was tested, which may not have been optimal for all targets. Finally, this study focuses on the ECoG signals, and our conclusion is therefore limited to cortical activity. Future studies should explore a broader range of frequencies and systematically assess motor symptoms to better characterize the relationship between FTG activity, DBS, and therapeutic benefit. Simultaneous LFPs and ECoG recordings could also help understand the changes induced by DBS on the network.

In summary, STN and GPi DBS modulated M1 NBG activity and improved motor symptoms, while GPe DBS did not. The differential modulation of NBG between therapeutic and nontherapeutic stimulation supports its potential as a biomarker for physiology-guided DBS. However, the lack of entrainment observed during high-frequency stimulation highlights the importance of tailoring stimulation parameters to the physiological properties of cortical oscillations. Further investigation is needed to determine the optimal conditions under which FTG can be observed and used as a reliable marker of therapeutic efficacy in neuromodulation.

## Data Availability

The deidentified raw data supporting the conclusions of this article will be made available by the authors, without undue reservation.
